# Socio-emotional competency matrix in nursing education: undergraduate student perspectives

**DOI:** 10.1590/1518-8345.7489.4482

**Published:** 2025-02-17

**Authors:** Laura Andrian Leal, Silvia Helena Henriques, Paulo Jorge Marcos Cruchinho, Iasmin Gabrielli da Silva, Josué Souza Gleriano, Carolina Cassiano

**Affiliations:** 1 Universidade de São Paulo, Escola de Enfermagem de Ribeirão Preto, PAHO/WHO Collaborating Centre for Nursing Research Development, Ribeirão Preto, SP, Brazil; 2 Centro de Investigação, Inovação e Desenvolvimento em Enfermagem de Lisboa (CIDNUR), Lisboa, Portugal; 3 Escola Superior de Enfermagem de Lisboa, Lisboa, Lisboa, Portugal; 4 Universidade do Estado do Mato Grosso, Enfermagem, Mato Grosso, MT, Brazil; 5 Scholarship holder at the Conselho Nacional de Desenvolvimento Científico e Tecnológico (CNPq), Brazil

**Keywords:** Nurses, Male, Education, Nursing, Graduate, Professional Competence, Emotions, Health Services, Social Skills

## Abstract

to develop a socioemotional competency matrix proposal for undergraduate nursing students.

this exploratory, descriptive study used a qualitative approach. Fifty-seven nursing students from a public higher education institution participated. Data were collected through focus groups and analyzed using inductive thematic analysis.

six socioemotional competencies were identified: Assertive Communication, Receptivity, Adaptability, Teamwork, Emotional Intelligence, and Shared Leadership. Additionally, recognizing associated behaviors/attitudes enabled the construction of the matrix. Strategies for competency development were mentioned, such as teacher support, participation in academic leagues, research projects, and workshops as initiatives from graduate programs.

the socio-emotional competency matrix for nursing students should assist health care managers and training centers in designing competency-based educational projects.

## Highlights

**(1)** The matrix should assist training centers in developing educational projects. **(2)** The matrix aims to address gaps in the training of future nurses. **(3)** The socio-emotional competency matrix for nursing contributes to qualification improvement.

## Introduction

Nursing is recognized as a complex field where professionals have direct and continuous contact with patients. Nurses play a crucial role across various areas within the health care system, contributing significantly to health promotion, disease prevention, treatment, and patient rehabilitation. Their work spans multiple settings, such as hospitals, primary health care, mental health, home care, as well as intensive care and emergency units. Given this diversity of practice environments, nurses maintain close relationships with both the multidisciplinary team and patients, performing a range of functions that contribute to holistic care and the well-being of individuals^(1-2)^.

Such proximity and contact between nurses, patients, and the care team can lead to emotional challenges for professionals, as the work process can be demanding, prolonged, and emotionally burdensome^(1)^. Given the range of nursing activities, it is essential to provide education that includes individual aspects, such as Socio-emotional Competencies (SEC), focusing on emotional regulation and interpersonal relationships.

In this context, the term competency is holistic, encompassing knowledge, skills, attitudes, and values; it primarily involves mobilizing values to address complex social and professional demands^(3)^. SEC are individual attributes that stem from biological predispositions and environmental factors, characterized by: 1) consistent patterns of thought, feeling, and action; 2) being acquired and developed through both formal and informal learning experiences; and 3) potentially influencing individuals’ socio-professional outcomes throughout their lives^(4)^; thus shaping the concept for this study.

Internationally, SEC have been linked to the capacity to successfully apply emotional intelligence concepts in daily practice to effectively lead and influence individuals and groups. They also encompass attributes such as self-awareness, humility, resilience, optimism, among others. Furthermore, like other competencies, SEC can be developed over time^(5-6)^.

In this line of thought, considering the professional praxis of nurses, researchers identify SEC as those most in need of development and proficiency, as this professional category deals with multiple functions in daily work, incidents, burnout issues, and the need to adapt to institutional standards. Additionally, ethics, moral principles, active listening, tolerance, and relationship aspects are cited as the least prevalent SEC in the hospital settings^(7)^.

From this perspective, SEC-based education can serve as a strategy to address the transformations in the labor market, particularly those related to emotions and mental health, which have come to the forefront.

Recent literature has shown that nursing professionals and students face personal dilemmas stemming from a lack of self-awareness, self-image, and difficulties in managing emotions and interpersonal relationships effectively, which can pose risks in the workplace due to challenges in handling frustrations, especially in the current post-pandemic context^(8-9)^, leading to potentially unsafe practices in the field.

This study addresses a significant gap in scientific knowledge by identifying SEC for undergraduate nursing students, especially through the development of a matrix to present these competencies. This research raises the following questions: What socio-emotional competencies are relevant in the education of nursing undergraduates? What behaviors/attitudes may be associated with these competencies?

It is believed that the answers to these questions will enable the construction of an SEC matrix that includes the behaviors/attitudes associated with these competencies, helping to establish the necessary profile of a nursing professional capable of managing interactions and emotions within their work praxis. A competency matrix is considered a foundational element for developing a competency-based curriculum, which should be built collectively with the academic community^(10)^.

Thus, this study aimed to develop a proposal for an SEC matrix for undergraduate nursing students.

## Method

### Type of study

This is an exploratory, descriptive, qualitative study. The guidelines of the Consolidated Criteria for Reporting Qualitative Research (COREQ)^(11)^ guided the writing of this article, derived from the conducted investigation.

### Study setting

The study was conducted at a Brazilian public higher education institution (IES) located in the city of Ribeirão Preto, São Paulo, Brazil. The IES is a public nursing university that offers two types of programs: a Bachelor’s degree and a combined Bachelor’s and Teaching degree. Both programs graduate around 130 students annually, with 80 students from the Bachelor’s in Nursing program and 50 from the combined Bachelor’s and Teaching degree program. At this institution, the Bachelor’s program lasts four years, comprising eight semesters, while the combined Bachelor’s and Teaching degree program spans five years, totaling 10 semesters.

### Population and selection criteria

The study participants were students in the final year of both listed programs and/or those who had already completed the Hospital Nursing Management course offered in both programs, in the 7th and 8th semesters for the Bachelor’s degree, and in the 9th and 10th semesters for the combined Bachelor’s and Teaching degree. This selection was based on the students’ greater knowledge base, their experience with administration and management internships (which address this competency), and, by this stage, their familiarity with the SEC necessary for nursing practice. Students who were on leave or had an inactive enrollment status were excluded from the study.

### Period

Data collection took place from March to September 2023.

### Data collection

Data were obtained through a questionnaire gathering socio-demographic information, including graduation period, gender, origin, and type of program. Subsequently, a script with guiding questions was used, employing the Focus Group (FG) technique, as outlined by Gatti^(12)^.

A purposive, judgment-based non-probability sampling technique was used to select participants for data collection. Additionally, the Snowball sampling technique^(13)^ was employed, involving the target audience, which consisted of nursing students. Participants were formally invited in person with an invitation letter (after class hours) or electronically to schedule the research activity and participate in Focus Groups (FG) according to their availability of day, time, and location, aiming to identify the SEC.

The FG were conducted face-to-face, at random, according to the offering of the Hospital Management course across different classes and programs, moderated by a researcher with a PhD in sciences and observed by a nursing graduate with a master’s degree in psychology and a PhD in sciences. Both the moderator and the observer have experience and training in the focus group technique and are affiliated with the same educational institution; thus, there are no conflicts of interest. The moderator, the first author of the study, was a postdoctoral student at the institution, and the observer, a PhD graduate, meaning that neither of them is a faculty member of the training institution. To minimize authority influence in the research due to academic titles, a welcoming environment was established, allowing autonomy in responses and harmony within the FGs to ensure genuinely voluntary and unbiased decisions and responses despite the presence of authority figures. Some strategies to limit this influence and ensure the ethical integrity of the research included: Anonymity and Confidentiality; Independent Informed Consent; Separation between Researcher and Evaluator; Voluntariness Statement; Neutral Environments, and adherence to ethics. These practices help mitigate authority influence and foster a more ethical, fair research environment that respects student autonomy.

The date and time for data collection were scheduled based on participants’ availability, and prior to the FG, a rapport was established with them through the introduction of the moderator and observer, including their academic and professional backgrounds and the reasons for conducting the research.

The sessions were audio-recorded, with each group lasting an average of 60 minutes. Before getting the FG started, a brief thematic explanation was provided on the definition of SEC according to the framework, clarifying any doubts regarding the aspects involved^(4)^. The guiding questions for the FGs were: “What socio-emotional competencies do you believe are necessary for your education?”; “What behaviors/attitudes are associated with these competencies?”; “Do you feel that the university has employed strategies to develop these competencies in you?”

These questions were developed by the researchers themselves, who have expertise in the topic. A field diary was also used to document the researchers’ observations throughout the groups. These notes, written during and immediately after the focus groups, proved invaluable for data analysis.

The exact number of participants and the quantity of FG conducted were determined based on the point of information power, and data collection concluded upon achieving the study’s objective^(14)^. It was not necessary to repeat the FG with the same participants.

Regarding data collection, all final-year students who were either currently enrolled in or had completed the Hospital Management course were invited to participate in the study. Of these, 57 (51.81%) students participated, 49 (44.54%) declined due to scheduling conflicts, and four (3.63%) declined without an apparent reason.

### Data analysis

The discussions from the FGs were manually transcribed and interpreted using inductive thematic analysis following these steps: transcription and reading of the data; systematic coding of interesting characteristics across the entire dataset; searching for themes by grouping codes; reviewing and verifying themes based on their corresponding codes; analyzing to refine the details of each theme; and finally, conducting a final analysis of selected excerpts, relating them to research guiding questions and literature, culminating in an academic report of the analysis^(15)^.

The analysis phase was conducted by the lead researcher, who has the education, training, and experience in the aforementioned method, which contributed to the reflexivity of the findings. The entire analysis was carefully reviewed and validated by the other authors of this study.

### Ethical aspects

This study was conducted in compliance with Resolution 466/12 and approved by the Research Ethics Committee (REC) of the Proposing Institution, under opinion no. 5.803.350 of 2022. Participants signed the Informed Consent Form (ICF), and confidentiality of responses was ensured.

The data obtained will be stored for five years (2023-2027), with access exclusively authorized for the responsible researchers and the REC of the Proposing Institution, if requested. After this period, all records will be deleted to prevent any form of participant identification.

To preserve participant anonymity, statements were presented with the Arabic number of the corresponding FG in the order it was conducted (FG1, FG2...). The citation of statements was structured as follows: (FG1. Student 1...) and so on.

## Results

Of the invited students, 57 (51.81%) participated in the study, of whom 51 (89.47%) were female and six (10.52%) were male. Ages ranged from 20 to 31 years old, with an average age of 23.01 years. The most prevalent city was Ribeirão Preto, SP, with 12 (21.05%) students.

Regarding the program, 41 (71.92%) students were from the Bachelor’s in Nursing program; 20 (48.78%) were in the 7th semester and 21 (51.21%) in the 8th semester, while 16 (28.07%) students were from the combined Bachelor’s and Teaching program; 13 (81.25%) in the 9th semester and three (18.75%) in the 10th semester.

Nine FG were conducted, eight of which included six students, and one FG included nine students. Following the FGs, analysis of participants’ statements, and the addition of theoretical references^(4)^, it was possible to characterize the SEC identified by undergraduate nursing students, and from these data, build a matrix of these competencies addressing their concepts and associated behaviors/attitudes. Two categories were formed: 1. Socio-emotional Competencies; 2. Strategies for Developing Socio-emotional Competencies. The first category, SEC, includes: Assertive Communication, Receptivity, Adaptability, Teamwork, Emotional Intelligence, and Shared Leadership, as illustrated in the following statements.

### Category 1: Socio-emotional Competencies

*Active listening, receptivity, empathy, truly hearing what the team is saying, being able to meet that need, and understanding what the team is trying to convey. Assertive communication and knowing how to handle frustrations...* (FG 1. Student 1)

*Teamwork, right? For me, it is a very emotional part because if we do not know how to work in a team, it will frustrate our emotions, and knowing how to adapt in the work environment.* (FG 6. Student 3)

*In addition, we need to have emotional intelligence, control of our emotions, and the ability to separate professionals from personal matters...* (FG 6. Student 2)

*To develop SEC, a nurse must be a democratic leader, know how to prioritize environmental issues, delegate responsibilities, organize dynamics, and for that, clear communication is essential.* (FG 6. Student 4)

**Figure 1 -:**
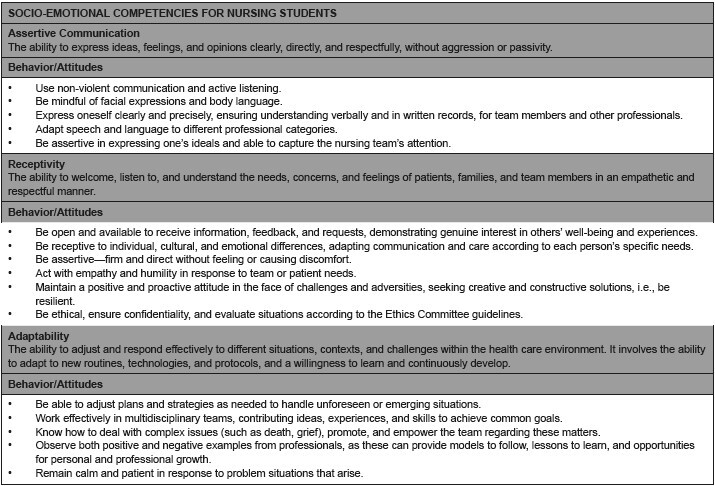
Socio-emotional Competencies Matrix with corresponding behaviors/attitudes. Ribeirão Preto, SP, Brazil, 2024

**Figure 2 -:**
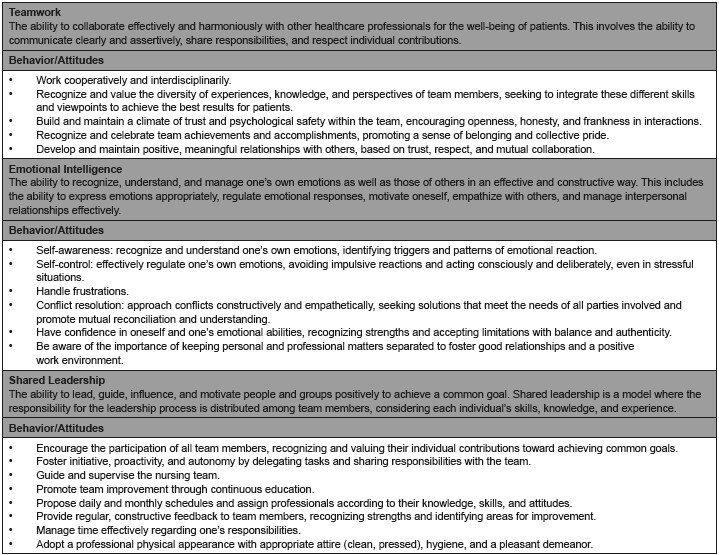
Socio-emotional Competencies Matrix with corresponding behaviors/attitudes. Ribeirão Preto, SP, Brazil, 2024

The SEC matrix for undergraduate nursing students is presented next, segmented in Figures 1 and 2. The matrix, in its composition, reveals the concepts of SEC and their respective behaviors/attitudes.

As additional results of this study, during the FG, students mentioned strategies that support the development of SEC: the support and mediation provided by faculty members and the IES, especially extracurricular initiatives, such as research projects or workshops linked to graduate-level initiatives.

### Category 2: Strategies for Developing Socio-emotional Competencies

*A lot is said about patients, but helping the professional is difficult.* (FG 1. Student 2)

*Emotional support from a few faculty members, support during the internship; when I went through a difficult time, the instructor guided me well.* (FG 7. Student 5)

*The strategy of using simulations was great; in all of them, we had debriefing sessions, feedback from professors, and that already contributed to developing our stance on the competencies we are discussing here... the debriefings are very valuable.* (FG 4. Student 2)

*We had a strategy outside the academic environment, CBT [Cognitive Behavioral Therapy] offered by a PhD student to address our emotional side and other issues like family distance or coping with separation during college; we would talk about this as a group and develop those skills together.* (FG 3. Student 1)

*We have more strategies in leagues and lectures. In PET [Tutorial Education Program], there are courses to develop conflict and management skills in discussion groups and on topics related to suicide prevention. Here at the university, there is also swimming and access to psychologists, but it is harder to get a spot.* (FG 2. Student 1)

## Discussion

The results enabled the creation of SEC matrix with associated behaviors/attitudes. A professional competency matrix can serve as a valuable management tool, helping to optimize human resource use by better aligning individuals with their roles, enhancing professional skills, and ultimately ensuring high-quality service delivery^(10)^.

In this regard, when it comes to SEC in health care services, nurses play a major role in coordinating the work of the multidisciplinary team. This coordination may occur through various competencies, including communication, which should be open and transparent, as well as receptivity, adaptability, leadership, and others, as reported in the findings of our study^(16)^.

Communication in health care, one of the highlighted competencies, is considered essential for establishing an interpersonal relationship between professionals and users, especially in relation to health promotion, disease prevention, and treatment^(17)^. Thus, assertive communication, as presented in the matrix, contributes to building positive and strong relationships, promoting user well-being, and enhancing team effectiveness in health care.

Nurses need to convey information clearly and comprehensibly while remaining receptive to users’ concerns and questions^(18)^, which requires a high level of SEC, including the ability to listen attentively and adjust communication to the emotional needs of the patient.

In this context, it is important to highlight the relationship between communication and its associated behaviors/attitudes, particularly the attention to facial expressions and/or non-verbal language. Researchers have demonstrated that it is possible to perceive another person’s emotions through facial expressions and changes in skin tone, such as blushing and gestures, for example. Inappropriate facial expressions can affect behavior in various ways, as non-verbal communication is noticeable in relationships and influences how people are perceived. Consequently, this can have a significant impact on people’s behavior and social interactions^(19)^.

By employing active listening, empathy, and assertive communication, receptivity naturally comes into play. Emotionally intelligent nurses are better communicators, exhibit more caring behaviors^(20)^ and possess adaptive skills for diverse situations^(21)^.

Along these lines, receptivity and adaptability are intrinsically linked to SEC, as both are essential for effectively managing social interactions, challenges, and changes in the work environment and personal life. Therefore, developing these competencies is necessary for effective social interactions and emotional well-being.

The health care work environment demands receptivity and adaptation to new challenges, and nurses continuously make important decisions for patient care. Receptivity occurs when professionals establish therapeutic bonds, promote trust and collaboration, and ensure patient- and family-centered health care adaptability is demonstrated when professionals are able to adjust to changes in a balanced manner. This involves managing uncertainties and adversities, which requires emotional control^(22-24)^.

Ethical behavior is essential for receptivity. Ethics is a fundamental attitude linked to nurses’ SEC in several ways. Therefore, beyond interpersonal abilities, SEC also encompasses how professionals relate to others, make decisions, and demonstrate respect for users.

Ethical conduct in nursing is defined as the ability to collaboratively build work processes, make ethical decisions, value solidarity, listen and share decisions, and manage conflicts by identifying their determinants^(24)^. In this regard, ethical principles and values should guide the actions and decisions of nursing professionals, ensuring the promotion, protection, recovery, and rehabilitation of people’s health^(25)^.

A receptive nurse must also possess empathy. Empathy enhances sensitivity to social cues that indicate others’ needs, and this competency is particularly important in caregiving professions, such as nursing^(26)^.

Regarding adaptability, the nurse should remain calm and patient when dealing with the various challenges in their work process. In this context, it is important to note that by acting with calmness, tolerance, respect for the team, tranquility, humility, and confidentiality (trustworthiness), the professional supports the delivery of physical care, emotional support, and the overall well-being of patients. Moreover, empathy, respect, calmness, and patience are considered critical components in establishing a relationship of trust between the nursing team, the patient, and the nurse^(27-28)^.

Another competency listed in the matrix was teamwork, which refers to an approach in which individuals collaborate and share responsibilities to achieve common goals. Scholars also note that for a group to transform into a team, certain factors are required, such as commitment, inter-cooperation, and a greater understanding of teamwork^(29-30)^.

In this context, teamwork consists of a strategy for integrating specialties and multiple professions, and it is essential for the development of comprehensive patient care. The attributes for this work are interconnected with interprofessional communication, recognition of other professionals, and the team’s common goals in the interdependence of actions, patient-centered care, and emotional intelligence^(31)^.

For nurses, emotional intelligence is fundamental, as it directly influences the quality of care provided to patients, the ability to work in a team, and the management of stressful or challenging situations in the workplace. The integration of emotional intelligence as an SEC provides a deep understanding of one’s own emotions and those of others, enabling the nurse to connect with patients, understand their emotional needs, and provide professional support throughout the treatment and recovery process^(32)^.

In this line of thought, other researchers have highlighted the relationship between this competency and professional behavior, as it integrates a moment of promoting emotional competency. Those who possess the ability to identify emotions, use them in the thought process, understand them, analyze them, and effectively manage them both in themselves and in relation to others are considered emotionally intelligent individuals. Emotional competency results from self-awareness and self-perception of efficacy in controlling situations^(28)^.

The behaviors/attitudes related to socioemotional intelligence are associated with distinguishing professional from personal matters, self-awareness, self-control, and the ability to manage frustrations and conflicts. Nursing work is known to be complex, involving bonds and close relationships with patients, the team, and family members; therefore, it is essential for the professional to prevent work from interfering with personal life. Additionally, it is crucial to manage conflicts constructively and empathetically.

In this regard, conflict management is another relevant factor for the success of a health care team, as it can directly impact the quality of care provided. Conflicts are understood as inherent situations in human life and can serve as constructive elements for personal development. Bearing this in mind, it is up to the professional responsible to manage any conflicts that arise or may arise to keep the team motivated and avoid impacts on productivity^(33-34)^.

For assertive conflict management, the nurse should employ shared leadership, also identified as an essential competency in student training. Leadership involves knowing how to delegate tasks and responsibilities. In the nursing work process, the nurse manages the team, which can lead to task overload for the leader. Such overload can be alleviated if the manager knows how to delegate activities to team members, allowing them to resolve issues appropriately without violating organizational culture. In addition to providing greater availability for the manager, delegating tasks enables an assessment of team members’ potential and commitment, also building a relationship of trust^(19)^.

The competency of shared leadership involves behaviors/attitudes related to demeanor, time management, continuous education, and personnel distribution. In this regard, nurses’ physical posture and appearance are highly observed and necessary for this competency. The way nurses present themselves physically can affect how they are perceived by patients, coworkers, and the multidisciplinary team, which, in turn, can influence social and emotional interactions^(28)^. Therefore, it is important to raise awareness among professionals about adopting a pleasant appearance and demeanor, helpful behavior, and wearing clean and appropriate clothing for the work environment.

Another behavior associated with shared leadership is time management. The ability to manage time effectively improves productivity and contributes to healthy relationships and emotional balance^(35)^.

Following this thinking, scholars have found few strategies for developing SEC for nursing students. In this study’s findings, there was little to no guidance provided by faculty members or specialists on emotional control during the undergraduate nursing program. The program’s curriculum does not include content, courses, or activities addressing this topic.

During the research, the use of FG itself served as a strategy to emphasize the socioemotional approach with students, encouraging the development of SEC among participants. FG is a collaborative research method that involves participants in the active construction of knowledge, and its approach explores the interaction between language, subjectivity, and the social construction of reality. This indicates that simple and brief resources can be adopted in the undergraduate program to create processes for SEC development^(36)^.

In this way, it is observed that increasing the emotional intelligence of health care professionals should become an exponential concern. Focusing solely on technical competencies does not guarantee professional success for nurses, and with technological advancements, non-technical competencies will be the differentiating factor among health care professionals.

Creating a balanced, harmonious classroom environment that fosters the learning of values and disciplinary content has become a significant challenge, requiring teachers to possess various skills and competencies, such as SEC^(37)^.

Some specific strategies can be listed, such as: support groups for emotional issues; Tutorial Education Programs; encouragement through research; therapy; emotionally focused simulations; academic leagues; career planning tutorials, among others. These findings align with those of other researchers who have identified several strategies that promote the development of personal and social competencies, such as: reflective group learning; experiential activities; the deepening of emotional intelligence competencies; simulations; research; and others. Although the use of identified strategies promotes emotional intelligence, this process is not static and requires constant training and analysis of nursing practice to improve behavior through the enhancement of personal and social competencies^(38-39)^.

This study highlights the importance of outlining an SEC matrix with its associated factors, behaviors/attitudes, recognizing the need to integrate this topic into the academic projects of nursing training centers, whether at the undergraduate or postgraduate level (specialization programs and research-focused programs). Outlining an SEC matrix specifically for nurses is a significant initiative, given the recognized importance of emotional and social skills in nursing practice^(40)^.

This research is limited by having been conducted exclusively at a public IES, without including other training centers. Furthermore, this investigation involved only final-year nursing students. It is important for further studies to be developed to encompass other educational institutions, including private ones, as well as graduates, faculty members, or even other professional categories, in order to verify potential differences and/or to determine the extent to which these findings may be generalized.

This study presents an SEC matrix that can offer several significant contributions to the advancement of scientific knowledge in the field of nursing education and training, as well as to the integration of SEC into university curricula.

## Conclusion

In addressing the objective of this study, it was possible to construct an SEC matrix for undergraduate nursing students. For each identified competency, students are expected to exhibit specific behaviors/attitudes. Providing a matrix with this information for a professional category can benefit students in their formative qualification and, ultimately, the workers themselves.

Reflecting on how future nurses are trained and prepared in terms of social and emotional skills will be the key to developing their emotional intelligence. Reflecting on emotions enhances self-awareness. Additionally, by characterizing SEC based on the perceptions of future nurses, they can be better equipped to manage the work environment, promoting their well-being and that of their team. Investing in the socioemotional development of nursing students has the potential to raise the standard of care provided and prepare resilient professionals who are equipped to face the challenges of clinical practice with compassion and competence.

Therefore, constructing this SEC matrix for nursing students should assist training centers in developing competency-based educational projects. Furthermore, it has the potential to fill possible gaps in the professional development of future nurses, preparing them with an SEC profile also sought by healthcare organizations and patients.
