# Supplementary material for: Socio-emotional competency matrix in nursing education: undergraduate student perspectives
Source: Rev Lat Am Enfermagem. 2025 Feb 17;33:e4482. doi: 10.1590/1518-8345.7489.4482 (PMC11835007; doi:10.1590/1518-8345.7489.4482)
Supplement: Translation

# Translation: Matriz de competências socioemocionais no ensino de enfermagem: concepções de discentes de graduação

**DOI:** 10.1590/1518-8345.7489.4483

**Authors:** Laura Andrian Leal, Silvia Helena Henriques, Paulo Jorge Marcos Cruchinho, Iasmin Gabrielli da Silva, Josué Souza Gleriano, Carolina Cassiano

**Affiliations:** 1 Universidade de São Paulo, Escola de Enfermagem de Ribeirão Preto, Centro Colaborador da OPAS/OMS para o Desenvolvimento da Pesquisa em Enfermagem, Ribeirão Preto, SP, Brasil; 2 Centro de Investigação, Inovação e Desenvolvimento em Enfermagem de Lisboa (CIDNUR), Lisboa, Portugal; 3 Escola Superior de Enfermagem de Lisboa, Lisboa, Lisboa, Portugal; 4 Universidade do Estado do Mato Grosso, Enfermagem, Mato Grosso, MT, Brasil; 5 Bolsista do Conselho Nacional de Desenvolvimento Científico e Tecnológico (CNPq), Brasil

**Keywords:** Enfermeiros, Educação em Enfermagem, Competência Profissional, Emoções, Serviços de Saúde, Habilidades Sociais

## Abstract

construir uma proposta de matriz de competências socioemocionais para discentes de graduação em enfermagem.

estudo exploratório, descritivo com abordagem qualitativa. Participaram 57 discentes de enfermagem de uma Instituição de Ensino Superior pública. Utilizou-se a técnica de grupo focal e para interpretação dos dados, a análise temática indutiva.

seis competências socioemocionais foram identificadas: Comunicação Assertiva; Receptividade; Adaptabilidade; Trabalho em Equipe; Inteligência Emocional e Liderança Compartilhada. Além disso, o reconhecimento de comportamentos/atitudes associados tornou possível construir uma matriz. Estratégias para o desenvolvimento das competências foram citadas, como: acolhimento dos docentes, participação em ligas acadêmicas, projetos de pesquisa e oficinas como iniciativas da pós-graduação.

a matriz de competências socioemocionais para discentes de enfermagem deve auxiliar gestores de saúde e centros formadores na elaboração de projetos pedagógicos orientados para formação baseada em competências.

## Destaques

**(1)** A matriz deve auxiliar centros formadores na elaboração de projetos pedagógicos. **(2)** A matriz deve suprir lacunas na formação dos futuros enfermeiros. **(3)** A matriz de competências socioemocionais de enfermagem auxilia na qualificação.

## Introdução

A enfermagem é reconhecida como uma área complexa, onde os profissionais têm contato direto e contínuo com os pacientes. O trabalho do enfermeiro é fundamental em diversos campos de atuação dentro do sistema de saúde, relevante para a promoção da saúde, prevenção de doenças, o tratamento e a reabilitação dos pacientes. Seu trabalho abrange diversos campos, como hospitais, atenção primária à saúde, saúde mental, cuidados domiciliares e unidades de terapia intensiva e emergência. Diante desta variedade de locais de atuação, os enfermeiros têm proximidade íntima tanto com a equipe multiprofissional quanto com os pacientes, e desempenham várias funções que contribuem para o cuidado holístico e o bem-estar dos indivíduos^(1-2)^.

Tal proximidade e contato do enfermeiro com os pacientes e a equipe de assistência podem desencadear problemas emocionais nos profissionais, já que o processo de trabalho pode ser árduo, demorado e doloroso^(1)^. Diante da gama de atividades de enfermagem, é imprescindível um ensino instrumentalizado em aspectos individuais, como as Competências Socioemocionais (CSE), ou seja, uma formação que tenha a preocupação com o controle das emoções e com as relações.

Neste contexto, o termo competência é holístico, e inclui conhecimentos, habilidades, atitudes e valores; implica sobretudo mobilizar os valores para responder às complexas demandas sociais e profissionais^(3)^. As CSE são atributos individuais que se originam de predisposições biológicas e fatores ambientais, e possuem características como: 1) padrões consistentes de pensamento, sentimento e ação; 2) são adquiridas e desenvolvidas a partir de experiências formais e informais de aprendizagem; e 3) influenciam potencialmente os resultados socioprofissionais dos indivíduos ao longo de suas vidas^(4)^, delineando o conceito para este estudo.

Em âmbito internacional, as CSE foram associadas com a capacidade de aplicar com sucesso os conceitos de inteligência emocional na prática diária para liderar e influenciar indivíduos e grupos de forma efetiva. Também incluem atributos como autoconsciência, humildade, resiliência, otimismo, entre outros. Ademais, como acontece com outras competências, as CSE podem ser desenvolvidas ao longo do tempo^(5-6)^.

Nessa linha de pensamento, considerando a práxis profissional do enfermeiro, pesquisadores apontam as CSE como as que mais carecem de desenvolvimento e aptidão, pois esta categoria profissional lida com múltiplas funções no cotidiano do trabalho, intercorrências, problemas de esgotamento, e necessidade de adequação às normas institucionais. Ainda é possível dizer que a ética e a moral, saber ouvir, tolerância e os aspectos ligados ao relacionamento são as CSE menos presentes no setor hospitalar^(7)^.

Nesta perspectiva, o ensino baseado em CSE pode funcionar como estratégia para enfrentamento das transformações presentes no mundo do trabalho em que, particularmente, os aspectos ligados a emoções e saúde mental estão à tona.

Recentemente, a literatura tem revelado que profissionais e discentes de enfermagem enfrentam dilemas pessoais decorrentes da falta de autoconsciência, autoimagem e dificuldades em administrar emoções e relacionamentos interpessoais de forma eficaz, o que pode levar a riscos no ambiente de trabalho, pela dificuldade em lidar com frustrações, principalmente no atual contexto pós-pandemia^(8-9)^, resultando em práticas potencialmente inseguras no setor.

Este estudo se faz relevante frente à lacuna do conhecimento científico quanto à identificação de CSE para discentes de graduação em enfermagem, sobretudo por meio da construção da matriz para apresentação dessas CSE. Esta investigação apresenta os seguintes questionamentos: quais competências socioemocionais são relevantes na formação de graduandos de enfermagem? Quais comportamentos/atitudes podem estar associados a estas competências?

Acredita-se que as respostas a estes questionamentos permitirão construir uma matriz de CSE que contemple os comportamentos/atitudes associados a elas, auxiliando no estabelecimento do perfil necessário do profissional de enfermagem para lidar com interações/emoções em sua práxis trabalhadora. Uma matriz de competências é considerada um elemento estruturante para o desenvolvimento de um *curriculum* baseado em competências, que deve ser construído coletivamente com a comunidade acadêmica^(10)^.

Assim, este estudo teve como objetivo construir uma proposta de matriz de CSE para discentes de graduação em enfermagem.

## Método

### Tipo de estudo

Trata-se de um estudo do tipo exploratório, descritivo, qualitativo. As diretrizes do *Consolidated Criteria for Reporting Qualitative Research* (COREQ)^(11)^ nortearam a escrita deste artigo, proveniente da investigação realizada.

### Cenário do estudo

O cenário de estudo foi uma Instituição de Ensino Superior Pública (IES) brasileira, localizada na cidade de Ribeirão Preto, SP, Brasil. A IES é uma universidade pública de enfermagem que oferece dois tipos de cursos: bacharelado e bacharelado e licenciatura. Ambos os cursos certificam cerca de 130 egressos anualmente, sendo 80 discentes do curso de bacharelado em enfermagem e 50 discentes do curso de bacharelado e licenciatura em enfermagem. Na referida instituição, o curso de bacharelado tem quatro anos de duração, com oito semestres; e o curso de bacharelado e licenciatura possui cinco anos de duração, perfazendo 10 semestres.

### População e critérios de seleção

Os participantes do estudo foram os discentes que cursavam o último ano da graduação de ambos os cursos listados, e/ou já haviam finalizado a disciplina de Gestão em Enfermagem Hospitalar oferecida nos dois cursos - no 7º e 8º semestres para o bacharelado, e no 9º e 10º semestres para bacharelado e licenciatura. O motivo da escolha foi pela maior bagagem de conhecimentos dos discentes, a realização de estágios de administração e gestão (que abordam a competência) além de, neste período, já terem a experiência de aprendizagem e aproximação com as CSE necessárias ao trabalho do enfermeiro. Discentes com a matrícula trancada ou afastados foram excluídos da pesquisa.

### Período

A coleta de dados foi realizada nos meses de março a setembro de 2023.

### Coleta de dados

Os dados foram obtidos por meio de um questionário com informações sociodemográficas do período da graduação, gênero, procedência e tipo de curso; e posteriormente, através de um roteiro com questões norteadoras, utilizando a técnica de Grupo Focal (GF), conforme o referencial proposto por Gatti^(12)^.

A amostragem não probabilística intencional baseada em julgamento foi a técnica utilizada na seleção dos participantes para coleta de dados. Também foi empregada a técnica de amostragem *Snowball* ou bola de neve^(13)^, envolvendo o público-alvo, que consistia nos discentes de enfermagem. Os participantes foram formalmente convidados, pessoalmente, por carta-convite (após o período de aulas) ou via eletrônica, a fim de participar e agendar a atividade de pesquisa e a realização dos Grupos Focais (GF), conforme a sua disponibilidade de dia, horário e local, com intuito de identificar as CSE.

Os GF foram conduzidos de modo aleatório, presencial, de acordo com o oferecimento da disciplina de Gestão Hospitalar por turmas e cursos diferentes, com a modera*ção* de uma pesquisadora doutora em ciências, e a observa*ção por uma* graduada em enfermagem, com mestrado em psicologia e doutorado em ciências. Tanto a moderadora quanto a observadora têm experiência e treinamento na técnica de grupo focal e são filiadas à mesma instituição de ensino, logo, não há conflitos de interesse. A moderadora, primeira autora do estudo, foi aluna de pós-doutorado da instituição, e a observadora, egressa de doutorado, ou seja, não são docentes da instituição formadora*.* Para diminuir a influência de autoridade na pesquisa devido às titulações, foi estabelecido um ambiente acolhedor, com autonomia nos relatos e harmonia entre os GF para garantir decisões e respostas genuinamente voluntárias e não influenciadas pela presença de figuras de autoridade. Algumas estratégias para limitar essa influência e assegurar a integridade ética da pesquisa incluíram: Anonimato e Confidencialidade; Consentimento Informado Independente; Separação entre Pesquisador e Avaliador; Declaração de Voluntariedade; Ambientes Neutros e utilização da ética. Essas práticas auxiliam a mitigar a influência da autoridade e promovem um ambiente de pesquisa mais ético, justo e de respeito à autonomia dos estudantes.

A data e o horário da coleta de dados foram agendados com base na disponibilidade dos participantes e, antes do desenvolvimento dos GF, foi estabelecido um relacionamento com eles por meio da introdução da moderadora e observadora, bem como de suas formações acadêmicas e profissionais, e dos motivos para a condução da pesquisa.

As informações foram audiogravadas com gravador e cada grupo teve duração média de 60 minutos. Antes de iniciar os GF, foi realizada uma breve explanação temática sobre a definição de CSE conforme o referencial, esclarecendo dúvidas sobre os aspectos envolvidos^(4)^. As questões norteadoras da condução dos GF foram: “Quais competências socioemocionais vocês acreditam que sejam necessárias para a sua formação?”; “Quais são os comportamentos/atitudes associados a estas competências?”; “Vocês percebem se a universidade tem utilizado estratégias para desenvolver essas competências em vocês?”.

Estas perguntas foram construídas pelos próprios pesquisadores, com expertise na temática. Um diário de campo também foi utilizado para registrar as percepções das pesquisadoras no decorrer dos grupos. Essas informações, redigidas durante e imediatamente após os grupos focais, foram de grande valia para a análise de dados.

O número exato de participantes e a quantidade de GF realizados foram determinados com base no ponto de alcance do poder da informação, e o encerramento da coleta de dados se deu ao se atingir o objetivo do estudo^(14)^. N*ão* foi necessário repetir os GF com os mesmos participantes.

Sobre a coleta de dados, todos os discentes do último ano, cursando ou tendo finalizado a disciplina de Gestão Hospitalar, foram convidados a participar da pesquisa. Destes, 57 (51,81%) discentes participaram efetivamente, 49 (44,54%) recusaram por questões de disponibilidade de horário entre os encontros e quatro (3,63%) por não aceitarem participar da pesquisa sem motivo aparente.

### Análise dos dados

As discussões provenientes dos GF foram manualmente transcritas e interpretadas conforme análise temática indutiva seguindo as etapas de: transcrição e leitura dos dados; codificação sistemática das características interessantes em todo o conjunto de dados; pesquisa de tópicos agrupando códigos; revisão e verificação dos tópicos aos quais eles respondem para extração de codificação; análise para refinar os detalhes de cada tópico; e, por fim, condução de uma análise final de trechos selecionados, relacionados a questões de orientação de pesquisa e literatura, gerando relatório acadêmico da análise^(15)^.

A fase de análise foi realizada pela pesquisadora principal, com formação, treinamento e experiência no método supracitado. Este fato contribuiu para o processo de reflexividade dos achados. Toda a análise foi cuidadosamente revisada e validada pelos demais autores deste estudo.

### Aspectos éticos

Este estudo foi desenvolvido em conformidade com a Resolução 466/12, aprovado pelo Comitê de Ética em Pesquisa (CEP) da Instituição Proponente, parecer nº 5.803.350 de 2022. Os participantes assinaram o Termo de Consentimento Livre e Esclarecido (TCLE), sendo assegurado o sigilo das respostas.

Os dados obtidos serão arquivados por cinco anos (2023-2027) e o acesso será autorizado exclusivamente para os pesquisadores responsáveis e pelo CEP da Instituição Proponente, caso seja solicitado. Após este período, todos os registros supracitados serão apagados, de modo a evitar qualquer tipo de identificação dos participantes.

Para preservar o anonimato dos participantes, os depoimentos foram apresentados com o número arábico do GF correspondente à ordem em que foi realizado (GF1, GF2...). A citação dos depoimentos foi estruturada da seguinte forma: (GF1. Discente 1...) e assim sucessivamente.

## Resultados

Dos discentes convidados, 57 (51,81%) participaram do estudo, dos quais 51 (89,47%) eram do sexo feminino e seis (10,52%) do sexo masculino. A idade variou de 20 a 31 anos, com média de 23,01 anos. A cidade de maior prevalência foi Ribeirão Preto, SP, com 12 (21,05%) discentes.

Em relação ao curso, 41 (71,92%) discentes eram do curso de bacharelado em enfermagem; 20 (48,78%) do 7º semestre e 21 (51,21%) do 8º semestre, enquanto, 16 (28,07%) discentes eram do curso de bacharelado e licenciatura; 13 (81,25%) do 9º semestre e três (18,75%) do 10º semestre.

Nove GF foram realizados, sendo oito grupos com a participação de seis discentes e um GF com nove discentes. Após a realização dos GF e análise dos depoimentos dos participantes com apoio do referencial teórico^(4)^, foi possível caracterizar as CSE identificadas pelos discentes de graduação em enfermagem, e a partir destes dados, construir uma matriz dessas competências abordando seus conceitos e comportamento/atitudes associados. Duas categorias foram formadas: 1. As Competências Socioemocionais; 2. Estratégias para o Desenvolvimento de Competências Socioemocionais. A primeira categoria CSE inclui: Comunicação Assertiva; Receptividade; Adaptabilidade; Trabalho em Equipe; Inteligência Emocional e Liderança Compartilhada, conforme os depoimentos a seguir.

### Categoria 1: As Competências Socioemocionais

*Escuta ativa, receptividade, empatia, você realmente ouvir o que a equipe está falando, conseguir atender aquela necessidade, captar o que a equipe está querendo passar pra você. A comunicação assertiva, e saber lidar com frustrações...*(GF 1. Discente 1)

*Trabalho em equipe, né? Pra mim, é uma parte muito emocional porque se a gente não sabe trabalhar em equipe, isso vai frustrar o nosso emocional, e saber se adaptar no meio de trabalho.* (GF 6. Discente 3)

*Além disso, precisamos ter a inteligência emocional, controle das nossas emoções, saber separar o profissional do pessoal...* (GF 6. Discente 2)

*Para conseguir ter CSE, o enfermeiro deve ser um líder democrático e saber priorizar questões de ambiente, delegar responsabilidades, realização de dinâmicas de organização, e para isso, comunicação clara.* (GF 6. Discente 4)

Na sequência, é apresentada a matriz de CSE dos discentes de graduação em enfermagem segmentada nas Figuras 1 e 2. A matriz, em sua composição, revela conceitos das CSE e seus respectivos comportamentos/atitudes.

**Figura 1 -:** Matriz de competências socioemocionais (CSE) com seus comportamentos/atitudes. Ribeirão Preto, SP, Brasil, 2024

**Figura 2 -:** Matriz de competências socioemocionais (CSE) com seus comportamentos/atitudes. Ribeirão Preto, SP, Brasil, 2024

Ainda como resultados deste estudo, durante os GF, os discentes mencionaram estratégias que auxiliam no desenvolvimento de CSE: o acolhimento e mediação dos docentes e da IES, especialmente extragraduação, como projetos de pesquisa ou oficinas ligadas a iniciativas da pós-graduação.

### Categoria 2: Estratégias para o Desenvolvimento de Competências Socioemocionais

*Muito se fala só sobre paciente, mas para [ajudar] o profissional* é *difícil.* (GF 1. Discente 2)

*Apoio emocional de alguns poucos docentes, acolhimento no estágio, quando passei por algo difícil, a docente me conduziu bem.* (GF 7. Discente 5)

*A estratégia de usar simulação foi ótimo, todas que nós tivemos, tivemos momentos de* debriefing*,* feedbacks *de professores e nisso já é um desenvolvimento de como que deve ser nossa postura nessas competências que estamos abordando aqui... os* debriefings são muito interessantes. (GF 4. Discente 2)

*Tivemos uma estratégia fora do* [ambiente] *acad*ê*mico, TCC [Terapia Cognitivo-Comportamental] oferecida por uma aluna de doutorado para saber sobre nosso lado emocional e outras questões de distância de família, ou lidar com distâncias na graduação, a gente conversava em grupo sobre isso e ia desenvolvendo aquela habilidade junto.* (GF 3. Discente 1)

*Temos estratégia mais em ligas, palestras. No PET* [Programa de Educação Tutorial]*, tem curso para desenvolver habilidades de conflitos e gestão em rodas de conversa, e sobre grupos de suicídio. Aqui na universidade também tem natação, psicólogo, mas é mais difícil conseguir vaga.* (GF 2. Discente 1)

## Discussão

Os resultados permitiram traçar uma matriz de CSE e seus comportamentos/atitudes associados. Uma matriz de competências profissionais, enquanto instrumento de gestão, pode facilitar a maximização do aproveitamento dos recursos humanos, direcionando-os com mais êxito para as funções desempenhadas pelo aprimoramento das habilidades dos profissionais e, como consequência, assegurar a qualidade dos serviços oferecidos^(10)^.

A esse respeito, em se tratando das CSE nos serviços de saúde, o enfermeiro é um dos grandes responsáveis por articular o trabalho da equipe multidisciplinar, o que pode ocorrer por meio de várias competências, dentre elas a competência de comunicação, que deve ser aberta e transparente, de receptividade, de adaptabilidade, liderança e outras, como reportam os achados de nosso estudo^(16)^.

A comunicação em saúde, uma das competências destacadas, é considerada fundamental para criar uma relação interpessoal entre profissionais e usuários, especialmente quando relacionada à promoção de saúde, prevenção e tratamento de doenças^(17)^. Assim, uma comunicação assertiva, apresentada na matriz, contribui para construir relacionamentos positivos e sólidos, promover o bem-estar do usuário e a eficácia do trabalho em equipe na área da saúde.

Os enfermeiros precisam transmitir informações de forma clara e compreensível, ao mesmo tempo em que se apresentam receptivos às preocupações e questionamentos dos usuários^(18)^, o que exige um alto nível de CSE, incluindo a capacidade de ouvir atentamente e ajustar a comunicação *à*s necessidades emocionais do paciente.

Nesse sentido, é relevante destacar a relação da comunicação e seus comportamentos/atitudes, ou seja, sobre o cuidado com expressões faciais e/ou linguagem não verbal. Pesquisadores já demonstraram que é possível perceber as emoções de outra pessoa por meio de expressões faciais e alterações na pigmentação cutânea, como rubor e gestos, por exemplo. A expressão facial inapropriada pode afetar o comportamento de várias maneiras, pois a comunicação não verbal é notável nos relacionamentos com os outros e em como as pessoas são percebidas. Consequentemente, isso pode ter impacto significativo no comportamento das pessoas e nas interações sociais^(19)^.

Ao utilizar a escuta ativa, empatia e comunicação assertiva, é inerente discorrer sobre a receptividade. Enfermeiros emocionalmente inteligentes são melhores comunicadores, exibem mais comportamentos de cuidado^(20)^ e habilidades adaptativas para situações diversas^(21)^.

Nessa linha de pensamento, a receptividade e a adaptabilidade estão intrinsecamente ligadas às CSE, uma vez que ambas são fundamentais para lidar adequadamente com as interações sociais, desafios e mudanças no ambiente de trabalho e na vida pessoal. Portanto, é necessário desenvolver essas competências para interações sociais eficazes e bem-estar emocional.

O ambiente de trabalho em saúde demanda receptividade e adaptação frente a novos desafios, e a todo instante, enfermeiros tomam decisões importantes para o cuidado. Assim, a receptividade acontece quando o profissional estabelece vínculos terapêuticos, promove a confiança e a colaboração, garantindo uma assistência de saúde centrada no paciente e na família; já a adaptabilidade *é* observada quando o profissional tem capacidade de se ajustar às mudanças de maneira equilibrada. Isso significa lidar com incertezas e adversidades, o que requer controle emocional^(22-24)^.

O comportamento ético é imprescindível para a receptividade. A ética é uma atitude fundamental ligada às CSE de enfermeiros e profissionais de saúde de várias maneiras. Portanto, além de capacidades interpessoais, as CSE também abarcam como os profissionais se relacionam com os outros, como tomam decisões e demonstram respeito pelos usuários.

A postura ética no trabalho do enfermeiro é conceituada como a capacidade de construir coletivamente processos de trabalho, decidir eticamente, valorizar a solidariedade, ouvir e compartilhar decisões e gerenciar conflitos, identificando seus determinantes^(24)^. Nesse sentido, os princípios e valores éticos devem nortear as ações e decisões dos profissionais de enfermagem, assegurando a promoção, proteção, recuperação e reabilitação da saúde das pessoas^(25)^.

O enfermeiro, ao ser receptivo, deve também possuir empatia. A empatia melhora a sensibilidade aos sinais sociais que indicam as necessidades dos outros, e esta competência é particularmente importante em profissões que envolvem a prestação de cuidados, como a de enfermeiro^(26)^.

Na adaptabilidade, o enfermeiro deve possuir calma e paciência ao lidar com as diferentes problemáticas do seu processo de trabalho. Nessa linha de pensamento, é importante observar que ao agir com calma, tolerância, respeito com a equipe, tranquilidade, humildade e sigilo (confiabilidade), o profissional corrobora com a prestação de cuidados físicos, apoio emocional e promoção do bem-estar geral dos usuários. Outrossim, a empatia, o respeito, a calma e a paciência são considerados componentes críticos para estabelecer uma relação de confiança entre equipe de enfermagem, usuário e o enfermeiro^(27-28)^.

Outra competência listada na matriz foi o trabalho em equipe, que se refere a uma abordagem na qual indivíduos colaboram e compartilham responsabilidades, com vistas a alcançar objetivos comuns. Estudiosos acrescentam ainda que, para um grupo se transformar em uma equipe, são necessários alguns fatores como comprometimento, intercooperação, e maior conhecimento sobre o trabalho em equipe^(29-30)^.

Neste contexto, o trabalho em equipe consiste em uma estratégia de integração das especialidades e múltiplas profissões, e é imprescindível para o desenvolvimento da assistência e do cuidado integral ao paciente. Os atributos para este trabalho estão interligados à comunicação interprofissional, ao reconhecimento dos demais profissionais, e aos objetivos comuns da equipe na interdependência das ações, na atenção centrada no paciente e na inteligência emocional^(31)^.

Assim, para o enfermeiro, a inteligência emocional é elementar, pois influencia diretamente a qualidade do cuidado prestado aos usuários, a capacidade de trabalhar em equipe e o gerenciamento de situações estressantes ou desafiadoras no ambiente de trabalho. A articulação da inteligência emocional como CSE fornece compreensão profunda das emoções próprias e dos outros, permite ao enfermeiro criar conexões com os usuários, entender suas necessidades emocionais e fornecer suporte profissional durante o processo de tratamento e recuperação^(32)^.

Nessa linha de pensamento, outros pesquisadores elencaram a relação dessa competência com o comportamento de profissionalismo, por integrar um momento de promoção da competência emocional. Aqueles que possuem a capacidade de identificar emoções, usá-las no processo de pensamento, entendê-las, analisá-las, e lidar com elas efetivamente em relação a si mesmos e aos outros, são indivíduos emocionalmente inteligentes. A competência emocional resulta do autoconhecimento e autopercepção da eficácia no controle das situações^(28)^.

Os comportamentos/atitudes ligados à inteligência socioemocional estão atrelados a distinguir o profissional do pessoal, ao autoconhecimento, autocontrole e a saber lidar com frustrações e conflitos. O trabalho do enfermeiro é conhecidamente complexo, envolve vínculo e proximidade com usuários, equipe e família, logo, é primordial que o profissional não deixe o trabalho interferir na sua vida pessoal. Além disso, é imprescindível saber gerenciar conflitos de maneira construtiva e empática.

Nesse sentido, a gestão de conflitos é outro fator relevante para o sucesso de uma equipe de saúde, pois pode afetar diretamente a qualidade do atendimento prestado. Os conflitos são entendidos como situações inerentes à vida dos seres humanos, e podem servir como algo construtivo para desenvolvimento pessoal. Tendo isso em vista, cabe ao profissional responsável gerenciar os conflitos que ocorram ou possam vir a ocorrer, com a finalidade de manter a equipe motivada e não causar impactos em sua produtividade^(33-34)^.

Para uma gestão de conflitos assertiva, o enfermeiro deve utilizar a liderança compartilhada, também identificada como competência essencial na formação do discente. A liderança pressupõe saber delegar tarefas e atividades. No processo de trabalho do enfermeiro, o mesmo gerencia a equipe, o que pode sobrecarregar de tarefas a pessoa no papel de líder. Tal sobrecarga pode ser amenizada se o gestor souber como delegar atividades para seus colaboradores, levando-os a resolver divergências de modo adequado, sem infringir a cultura organizacional. Além de proporcionar maior disponibilidade ao gestor, o comportamento de delegar tarefas permite analisar o potencial de seus colaboradores e seu comprometimento, construindo também uma relação de confiança^(19)^.

A competência da liderança compartilhada pressupõe comportamentos/atitudes relacionados à postura, gestão do tempo, educação permanente e distribuição de pessoal. Nesse sentido, a postura física e a aparência dos enfermeiros são muito observadas e necessárias para esta competência. A forma como os enfermeiros se apresentam fisicamente pode afetar a forma como são percebidos pelos usuários, colegas de trabalho e equipe multidisciplinar, o que, por sua vez, pode influenciar as interações sociais e emocionais^(28)^. Por isso, é importante conscientizar o profissional sobre a adoção de postura e aparência agradáveis, comportamento solícito, e o uso de vestimentas limpas e adequadas ao ambiente de trabalho.

Outro comportamento associado à liderança compartilhada consiste na gestão do tempo. Ter habilidade de gerenciar o tempo com efetividade melhora a produtividade e contribui para relacionamentos saudáveis e equilíbrio emocional^(35)^.

Nessa linha de pensamento, estudiosos encontraram poucas estratégias de desenvolvimento das CSE para discentes de enfermagem. Nos achados deste estudo, foi possível perceber pouca ou quase nenhuma orientação por parte de docentes ou profissionais especialistas na área a respeito do controle das emoções durante o curso de graduação em enfermagem. O currículo do curso não apresenta conteúdos, disciplinas ou atividades que apontem para este tema.

No desenvolvimento da pesquisa, o próprio uso de GF constituiu uma estratégia para valorizar a abordagem socioemocional com os discentes, provocando a mobilização das CSE entre os participantes. O GF é um método colaborativo de pesquisa que envolve participantes na construção ativa de conhecimento, e sua abordagem explora a interação entre a linguagem, a subjetividade e a construção social da realidade. Isso mostra que recursos simples e breves podem ser adotados no curso de graduação, visando criar processos de desenvolvimento de CSE^(36)^.

Desta forma, observa-se que o aumento da inteligência emocional dos profissionais de saúde deverá ser uma preocupação exponencial. O foco apenas nas competências técnicas não garante o sucesso profissional do enfermeiro, e com o aumento tecnológico, as competências não técnicas serão o fator diferenciador entre os profissionais de saúde.

A construção de um ambiente de sala de aula equilibrado, harmonioso e favorável à aprendizagem de valores e conteúdos disciplinares tem se tornado um grande desafio a cada dia e exige do professor conhecimentos e competências diversas, como é o caso das CSE^(37)^.

Algumas estratégias pontuais podem ser elencadas, como: grupos de apoio para questões emocionais; Programas de Educação Tutorial; incentivo por meio de pesquisas; terapia; simulações de cunho emocional; ligas acadêmicas; tutorias sobre planejamento de carreira, entre outros. Esses dados vão de encontro aos de outros pesquisadores que identificaram diversas estratégias promotoras do desenvolvimento de competências pessoais e sociais, tais como: a aprendizagem reflexiva em grupo; as atividades experienciais; o aprofundamento de competências de inteligência emocional; simulações; pesquisas; e outros. Embora a utilização das estratégias identificadas promova inteligência emocional, este processo não é estanque e requer treino e análise constantes da prática do enfermeiro, com vistas a melhorar o seu comportamento através do aperfeiçoamento das competências pessoais e sociais^(38-39)^.

Este trabalho define a importância de traçar uma matriz de CSE com seus fatores associados, comportamentos/atitudes, vislumbrando a necessidade de integrar esta temática nos projetos acadêmicos de centros formadores de enfermagem, seja na graduação, ou cursos de pós-graduação *lato* e *stricto sensu*. Traçar uma matriz de CSE específica para enfermeiros é uma iniciativa de grande importância, dada a reconhecida relevância das habilidades emocionais e sociais na prática da enfermagem^(40)^.

Esta pesquisa apresenta a limitação de ter sido realizada unicamente em uma IES pública, não abarcando a análise de demais centros formadores. Ademais, esta investigação foi realizada com a participação exclusiva de discentes de enfermagem do último ano. É importante que outros estudos sejam desenvolvidos, a fim de abranger outras instituições de ensino, inclusive privadas, além de profissionais graduados, docentes ou até mesmo outras categorias profissionais, com o intuito de verificar se há divergências e/ou para determinar a extensão da generalização das descobertas.

Este trabalho mostra uma matriz de CSE que pode oferecer várias contribuições significativas para o avanço do conhecimento científico no campo da educação e formação em enfermagem, bem como a integração de CSE no currículo acadêmico das universidades.

## Conclusão

Ao responder o objetivo deste estudo, foi possível construir uma matriz de CSE para o discente de graduação em enfermagem. Para cada competência identificada, o discente deve ter atitudes/comportamentos específicos. Disponibilizar uma matriz com essas informações de uma categoria profissional pode beneficiar os discentes em sua qualificação formativa e os próprios trabalhadores.

A reflexão sobre como ocorre a formação e preparo dos futuros enfermeiros em relação às aptidões sociais e emocionais será o diferencial para o desenvolvimento da inteligência emocional dessas pessoas. Pensar sobre as emoções melhora a capacidade de autoconhecimento. Além disso, ao caracterizar as CSE pela percepção de futuros enfermeiros, eles podem ser capacitados na gerência do ambiente de trabalho, promovendo seu próprio bem-estar e o de sua equipe. Investir no desenvolvimento socioemocional dos discentes de enfermagem tem potencial para elevar o padrão de cuidado oferecido e preparar profissionais resilientes e aptos a enfrentar os desafios da prática clínica com compaixão e competência. Portanto, a construção desta matriz sobre CSE para discentes de enfermagem deve auxiliar centros formadores na elaboração de projetos pedagógicos orientados para a formação baseada em competências. Outrossim, tem potencial para suprir possíveis lacunas quanto ao desenvolvimento profissional dos futuros enfermeiros, preparando-os com um perfil de CSE também almejado pelas organizações de saúde e pacientes assistidos.

# Translation: Matriz de competencias socioemocionales en la formación en enfermería: concepciones de estudiantes de grado

**DOI:** 10.1590/1518-8345.7489.4481

**Authors:** Laura Andrian Leal, Silvia Helena Henriques, Paulo Jorge Marcos Cruchinho, Iasmin Gabrielli da Silva, Josué Souza Gleriano, Carolina Cassiano

**Affiliations:** 1 Universidade de São Paulo, Escola de Enfermagem de Ribeirão Preto, Centro Colaborador de la OPS/OMS para el Desarrollo de la Investigación en Enfermería, Ribeirão Preto, SP, Brasil; 2 Centro de Investigação, Inovação e Desenvolvimento em Enfermagem de Lisboa (CIDNUR), Lisboa, Portugal; 3 Escola Superior de Enfermagem de Lisboa, Lisboa, Lisboa, Portugal; 4 Universidade do Estado do Mato Grosso, Enfermagem, Mato Grosso, MT, Brasil; 5 Becaria del Conselho Nacional de Desenvolvimento Científico e Tecnológico (CNPq), Brasil

**Keywords:** Enfermeros, Educación en Enfermería, Competencia Profesional, Emociones, Servicios de Salud, Habilidades Sociales

## Abstract

crear una propuesta de matriz de competencias socioemocionales para estudiantes de grado en enfermería.

estudio exploratorio, descriptivo con enfoque cualitativo. Participaron 57 estudiantes de enfermería de una Institución de Educación Superior pública. Se utilizó la técnica de grupo focal y para la interpretación de los datos se utilizó el análisis temático inductivo.

se identificaron seis competencias socioemocionales: Comunicación Asertiva; Receptividad; Adaptabilidad; Trabajo en equipo; Inteligencia Emocional y Liderazgo Compartido. Además, el reconocimiento de comportamientos/actitudes asociados permitió crear una matriz. Se mencionaron estrategias para desarrollar las competencias, tales como: recepción de los docentes, participar en ligas académicas, proyectos de investigación y talleres como iniciativas de posgrado.

la matriz de competencias socioemocionales para estudiantes de enfermería debe ayudar a los gestores de salud y a las instituciones de formación a desarrollar proyectos pedagógicos orientados a la formación basada en competencias.

## Destacados

**(1)** La matriz debe ayudar a las instituciones de formación a elaborar proyectos pedagógicos.**(2)** La matriz debe llenar vacíos en la formación de los futuros enfermeros. **(3)** La matriz de competencias socioemocionales de enfermería contribuye a la cualificación.

## Introducción

La enfermería es reconocida como un área compleja, en la que los profesionales tienen contacto directo y continuo con los pacientes. La labor del enfermero es fundamental en diversos campos de acción dentro del sistema de salud, es importante para la promoción de la salud, la prevención de enfermedades, el tratamiento y la rehabilitación de los pacientes. Su trabajo abarca diversos campos como hospitales, atención primaria de salud, salud mental, atención domiciliaria y unidades de cuidados intensivos y emergencias. Debido a la variedad de lugares en los que se desempeña, el enfermero tiene contacto estrecho tanto con el equipo multidisciplinario como con los pacientes, y desempeña diferentes funciones que contribuyen al cuidado holístico y al bienestar de las personas^(1-2)^.

Esa proximidad y el contacto entre el enfermero y los pacientes y el equipo de atención puede desencadenar problemas emocionales en dicho profesional, ya que el proceso de trabajo puede ser arduo, prolongado y doloroso^(1)^. Debido a la amplia gama de actividades que desempeña el enfermero, es fundamental que la enseñanza pueda dotarlos de herramientas a nivel individual, como las Competencias socioemocionales (CSE), es decir, que haya una formación que se ocupe del control de las emociones y las relaciones.

En este contexto, el término competencia es holístico e incluye conocimientos, habilidades, actitudes y valores; implica, sobre todo, movilizar los valores para responder a las complejas demandas sociales y profesionales^(3)^. Las CSE son atributos individuales que se adquieren gracias a predisposiciones biológicas y factores ambientales, y tienen características tales como: 1) patrones consistentes de pensamiento, sentimiento y acción; 2) se adquieren y desarrollan a partir de experiencias de aprendizaje formales e informales; y 3) afectan potencialmente los resultados socioprofesionales de los individuos a lo largo de su vida^(4)^; lo que perfila el concepto para este estudio.

A nivel internacional, las CSE se han asociado con la capacidad de aplicar con éxito los conceptos de inteligencia emocional en la práctica diaria para liderar e influir eficazmente en individuos y grupos. También incluyen atributos como el autoconocimiento, la humildad, la resiliencia, el optimismo, entre otros. Además, como ocurre con otras competencias, las CSE se puede desarrollar con el tiempo^(5-6)^.

Al considerar la praxis profesional de los enfermeros, los investigadores señalan que las CSE son las que requieren más desarrollo y aptitud, dado que dichos profesionales lidian en el trabajo diario con múltiples funciones, complicaciones, problemas de agotamiento y con la necesidad de adaptarse a las normas institucionales. Incluso se puede afirmar que la ética y la moral, el saber escuchar, la tolerancia y los aspectos vinculados a las relaciones son las CSE que menos se ven en el sector hospitalario^(7)^.

Por consiguiente, la enseñanza basada en las CSE puede funcionar como una estrategia para afrontar las transformaciones que ocurren en el mundo del trabajo, dado que los aspectos vinculados a las emociones y la salud mental, en particular, se manifiestan.

Recientemente, la literatura ha revelado que los profesionales y estudiantes de enfermería enfrentan dilemas personales derivados de la falta de autoconciencia, de autoimagen y de dificultades para gestionar eficazmente las emociones y las relaciones interpersonales, lo que puede generar riesgos en el ambiente laboral, debido a la dificultad que tienen para lidiar con la frustración, especialmente en el actual contexto pospandemia^(8-9)^, y pueden dar como resultado prácticas potencialmente inseguras en el sector.

Este estudio es importante debido al vacío que hay en el conocimiento científico sobre la identificación de las CSE para los estudiantes de grado en enfermería, sobre todo en lo que respecta a la elaboración de una matriz para presentar las CSE. En esta investigación se plantean las siguientes preguntas: ¿qué competencias socioemocionales son relevantes en la formación de estudiantes de enfermería? ¿Qué comportamientos/actitudes pueden asociarse con esas competencias?

Se cree que las respuestas a esas preguntas permitirán elaborar una matriz de CSE que incluya los comportamientos/actitudes asociados a las mismas y ayudarán a establecer el perfil que debe tener el profesional de enfermería para lidiar con las interacciones/emociones en la práctica laboral. La matriz de competencias se considera un elemento estructurante para el desarrollo de un currículo basado en competencias, que hay que elaborar colectivamente con la comunidad académica^(10)^.

Por lo tanto, este estudio tuvo como objetivo crear una propuesta de matriz de CSE para estudiantes de grado en enfermería.

## Método

### Tipo de estudio

Se trata de un estudio exploratorio, descriptivo y cualitativo. Para redactar este artículo, producto de la investigación realizada, se siguieron los lineamientos del *Consolidated Criteria for Reporting Qualitative Research* (COREQ)^(11)^.

### Escenario del estudio

El escenario del estudio fue una Institución de Educación Superior Pública (IES) brasileña, ubicada en la ciudad de Ribeirão Preto, SP, Brasil. La IES es una universidad pública de enfermería que dicta dos tipos de carrera: licenciatura y licenciatura y profesorado. Ambas carreras certifican alrededor de 130 egresados anualmente, 80 estudiantes de la carrera licenciatura en enfermería y 50 estudiantes de la carrera licenciatura y profesorado en enfermería. En esa institución, la carrera de licenciatura tiene una duración de cuatro años, con ocho semestres; y la carrera de licenciatura y profesorado tiene una duración de cinco años, 10 semestres en total.

### Población y criterios de selección

Los participantes del estudio eran estudiantes que cursaban el último año de las dos carreras de grado que dicta la institución, y/o ya habían cursado la asignatura Gestión de Enfermería Hospitalaria que se dicta en ambos cursos; en el 7º y 8º semestre de la licenciatura, y en el 9º y 10º semestres de la licenciatura y profesorado. Dicha elección se debe a que los estudiantes a esa altura de la carrera cuentan con una mayor base de conocimiento, realizan las pasantías en administración y gestión (que abordan la competencia) y porque en esa instancia ya cuentan con la experiencia de aprendizaje y están próximos a tener las CSE necesarias para llevar a cabo el trabajo de enfermero. Los estudiantes cuya matrícula no estaba vigente o no estaban cursando la carrera en ese momento fueron excluidos de la investigación.

### Periodo

La recolección de datos se realizó de marzo a septiembre de 2023.

### Recolección de datos

Los datos se obtuvieron a través de un cuestionario con información sociodemográfica sobre el período de la carrera de grado, género, procedencia y tipo de carrera; y posteriormente, mediante un cuestionario con preguntas orientadoras, utilizando la técnica de Grupo Focal (GF), según el marco propuesto por Gatti^(12)^.

La técnica utilizada para seleccionar a los participantes para la recopilación de datos fue muestreo no probabilístico intencional basado en juicios. También se utilizó la técnica de muestreo por *Snowball* o bola de nieve^(13)^, que involucra al público objetivo, que estuvo conformado por estudiantes de enfermería. Los participantes fueron contactados formalmente, de manera presencial, mediante carta de invitación (después del cursado de clases) o de forma electrónica, para participar y programar la actividad de investigación y la realización de Grupos Focales (GF), en función de su disponibilidad de día, hora y lugar, con el objetivo de identificar las CSE.

Los GF se realizaron de manera aleatoria, presencial, durante el dictado de la asignatura Gestión Hospitalaria en diferentes comisiones y carreras, con la moderación de una investigadora doctora en ciencias, y la observación de una licenciada en enfermería, con maestría en psicología y doctorado en ciencia. Tanto la moderadora como la observadora tienen experiencia y capacitación en la técnica de grupo focal y pertenecen a la misma institución educativa, por lo tanto, no hay conflicto de intereses. La moderadora, primera autora del estudio, era estudiante de posdoctorado de la institución, y la observadora, era egresada de doctorado, es decir, ninguna es profesora de la institución de formación. Para reducir la influencia de la autoridad en la investigación debido a los títulos*, se* creó un ambiente acogedor, con autonomía para los informes y armonía entre los GF para garantizar que las decisiones y respuestas fueran genuinamente voluntarias y no influenciadas por la presencia de figuras de autoridad. Algunas estrategias para limitar esa influencia y garantizar la integridad ética de la investigación incluyeron: Anonimato y Confidencialidad; Consentimiento Informado Independiente; Separación entre Investigador y Evaluador; Declaración de Voluntad; Ambientes Neutrales y uso de la ética. Estas prácticas ayudan a reducir la influencia de la autoridad y promover un entorno de investigación más ético y justo que respete la autonomía de los estudiantes.

La fecha y hora de recolección de datos se programaron en función de la disponibilidad de los participantes y, antes de realizar los GF, se estableció una relación con ellos al presentarles a la moderadora y la observadora, así como los antecedentes académicos y profesionales de las mismas, y los motivos de la investigación.

La información fue grabada en audio con una grabadora y cada grupo tuvo una duración promedio de 60 minutos. Antes de iniciar el GF, se dio una breve explicación temática sobre la definición de CSE según el marco teórico y se aclararon las dudas sobre los aspectos involucrados^(4)^. Las preguntas orientadoras para la realización del GF fueron: “¿Qué competencias socioemocionales creen que son necesarias para su formación?”; “¿Cuáles son los comportamientos/actitudes asociados con esas competencias?”; “¿Notaron si la universidad ha utilizado estrategias para que desarrollen esas competencias?”.

Dichas preguntas fueron elaboradas por las mismas investigadoras, expertas en el tema. También se utilizó un diario de campo para registrar las percepciones de las investigadoras durante la realización de los grupos. La información escrita durante e inmediatamente después de los grupos focales fue de gran ayuda para el análisis de datos.

El número exacto de participantes y la cantidad de GF realizados se determinaron en función del punto de alcance del poder de la información, y la recolección de datos finalizó cuando se logró el objetivo del estudio^(14)^. No fue necesario repetir los GF con los mismos participantes.

En cuanto a la recolección de datos, fueron invitados a participar de la investigación todos los estudiantes del último año, que cursaban o habían cursado la asignatura Gestión Hospitalaria. De ellos, 57 (51,81%) estudiantes participaron efectivamente, 49 (44,54%) se negaron por falta de disponibilidad horaria para las reuniones y cuatro (3,63%) no aceptaron participar en la investigación sin motivo aparente.

### Análisis de los datos

Las discusiones de los GF fueron transcritas manualmente e interpretadas mediante análisis temático inductivo siguiendo los pasos de: transcripción y lectura de datos; codificación sistemática de características interesantes en todo el conjunto de datos; búsqueda de tópicos agrupando códigos; revisión y verificación de los temas a los que responden para la extracción de codificación; análisis para refinar los detalles de cada tópico; y, por último, realización de un análisis final de extractos seleccionados, relacionados con las preguntas orientadoras de la investigación y la literatura, y se generó un informe académico del análisis^(15)^.

La fase de análisis fue realizada por la investigadora principal, con formación, capacitación y experiencia en el método antes mencionado. Este hecho contribuyó al proceso de reflexividad de los hallazgos. Todo el análisis fue revisado y validado cuidadosamente por los otros autores de este estudio.

### Aspectos éticos

Este estudio fue desarrollado de acuerdo con la Resolución 466/12, aprobada por el Comité de Ética en Investigación (CEI) de la Institución proponente, según dictamen nº 5.803.350 de 2022. Los participantes firmaron el Formulario de Consentimiento Libre e Informado (FCLI), y se garantizó la confidencialidad de las respuestas.

Los datos obtenidos serán archivados por cinco años (2023-2027) y el CEI de la Institución Proponente autorizará exclusivamente el acceso a los mismos a los investigadores responsables y solo si se lo solicitan. Transcurrido este plazo, todos los registros mencionados anteriormente serán eliminados, para evitar cualquier tipo de identificación de los participantes.

Para preservar el anonimato de los participantes, las declaraciones se presentaron con el número arábigo del GF correspondiente al orden en que fueron realizados (GF1, GF2...). La cita de los enunciados se estructuró de la siguiente manera: (GF1. Estudiante 1...) y así sucesivamente.

## Resultados

De los estudiantes invitados, participaron en el estudio 57 (51,81%), de los cuales 51 (89,47%) eran del sexo femenino y seis (10,52%) del sexo masculino. La edad osciló entre 20 y 31 años, con una media de 23,01 años. El municipio con mayor prevalencia fue Ribeirão Preto, SP, con 12 (21,05%) estudiantes.

En cuanto a la carrera, 41 (71,92%) estudiantes cursaban la licenciatura en enfermería; 20 (48,78%) el 7mo semestre y 21 (51,21%) el 8vo semestre, mientras que 16 (28,07%) estudiantes eran de la carrera licenciatura y profesorado; 13 (81,25%) cursaban el 9° semestre y tres (18,75%) el 10° semestre.

Se realizaron nueve GF, ocho grupos contaron con la participación de seis alumnos y un GF tenía nueve alumnos. Después de realizar el GF, analizar los discursos de los participantes y el marco teórico^(4)^, se pudieron caracterizar las CSE identificadas por los estudiantes de grado en enfermería, y a partir de esos datos, se elaboró una matriz de esas competencias abordando sus conceptos y comportamientos/actitudes asociados. Se conformaron dos categorías: 1. Competencias socioemocionales; 2. Estrategias para Desarrollar Competencias Socioemocionales. La primera categoría CSE incluye: Comunicación Asertiva; Receptividad; Adaptabilidad; Trabajo en equipo; Inteligencia Emocional y Liderazgo Compartido, según las siguientes declaraciones.

### Categoría 1: Competencias Socioemocionales

*Escucha activa, receptividad, empatía, realmente escuchar lo que dice el equipo, poder satisfacer esa necesidad, captar lo que el equipo intenta transmitirte. Comunicación asertiva, y saber lidiar con la frustraci*ón*...* (GF 1. Estudiante 1)

*Trabajo en equipo, ¿verdad? Para mí es una parte muy emocional porque si no sabemos trabajar en equipo nos vamos a frustrar emocionalmente y el saber adaptarnos al entorno laboral*. (GF 6. Estudiante 3)

*Además, necesitamos tener inteligencia emocional, control sobre nuestras emociones, saber separar lo profesional de lo personal...* (GF 6. Estudiante 2)

*Para lograr tener CSE, el enfermero debe ser un líder democrático y saber priorizar los temas del entorno, delegar responsabilidades, realizar dinámicas organizacionales y, para ello, tener una comunicación clara.* (GF 6. Estudiante 4)

A continuación, se presenta la matriz de CSE de los estudiantes de grado en enfermería, segmentada en las Figuras 1 y 2. La matriz, en su composición, revela conceptos de las CSE y sus respectivos comportamientos/actitudes.

**Figura 1 -:** Matriz de competencias socioemocionales (CSE) con sus comportamientos/actitudes. Ribeirão Preto, SP, Brasil, 2024

**Figura 2 -:** Matriz de competencias socioemocionales (CSE) con sus comportamientos/actitudes. Ribeirão Preto, SP, Brasil, 2024

Además, como resultados de este estudio, durante los GF, los estudiantes mencionaron estrategias que contribuyen al desarrollo de la CSE: la recepción e intermediación de los docentes y de la IES, especialmente en trabajos que no forman parte del cursado de la carrera, como proyectos de investigación o talleres vinculados a iniciativas de posgrado.

### Categoría 2: Estrategias para Desarrollar Competencias Socioemocionales

*Se habla mucho del paciente, pero [ayudar] el profesional es difícil.* (GF 1. Estudiante 2)

*Apoyo emocional de algunos docentes, apoyo durante la pasantía, cuando pasé por algo difícil, el docente me orient*ó bien. (GF 7. Estudiante 5)

*La estrategia de usar simulación estuvo genial, todas las que hicimos, tuvimos momentos de* debriefing*,* feedback *de los docentes y desarrollamos cuál debe ser nuestra postura en las competencias que estamos abordando... los* debriefings *son muy interesantes.* (GF 4. Estudiante 2)

*Hubo una estrategia fuera de lo académico [ambiente], TCC [Terapia Cognitivo-Conductual] dictada por una alumna de doctorado para conocer nuestro lado emocional y otros temas como el distanciamiento de la familiar, o lidiar con las distancias durante el cursado de la carrera, conversamos en grupo sobre eso e íbamos desarrollando esa habilidad juntos*. (GF 3. Estudiante 1)

*Realizamos estrategia más en ligas, conferencias. En el PET [Programa de Educación Tutorial] hay un curso para desarrollar habilidades de gestión y conflictos en círculos de conversación y en grupos de suicidio. Aquí en la universidad también hay natación y psicólogos, pero es más difícil conseguir cupo.* (GF 2. Estudiante 1)

## Discusión

Los resultados permitieron elaborar una matriz de CSE y sus comportamientos/actitudes asociados. Una matriz de competencias profesionales, como instrumento de gestión, puede facilitar la maximización del aprovechamiento de los recursos humanos y orientarlos con mayor éxito a las funciones que desempeñan, al mejorar las habilidades de los profesionales, y, por ende, garantizar la calidad de los servicios que brindan^(10)^.

En este sentido, cuando se trata de CSE en los servicios de salud, el enfermero es en gran medida responsable de coordinar el trabajo del equipo multidisciplinario, lo que puede realizar a través de varias competencias, entre ellas la capacidad de comunicación, que debe ser abierta y transparente, la receptividad, la adaptabilidad, el liderazgo y otras, como indican los hallazgos de nuestro estudio^(16)^.

La comunicación en salud, una de las habilidades destacadas, se considera fundamental para crear una relación interpersonal entre profesionales y pacientes, especialmente cuando se relaciona con la promoción de la salud, la prevención y el tratamiento de enfermedades^(17)^. Por ese motivo, la comunicación asertiva, presentada en la matriz, contribuye a construir relaciones positivas y sólidas y a promover el bienestar de los pacientes y la eficacia del trabajo en equipo en el campo de la salud.

El enfermero debe transmitir información de forma clara y comprensible, ser receptivo a las preocupaciones y preguntas de los pacientes^(18)^, lo que requiere un alto nivel de CSE, e incluye la capacidad de escuchar atentamente y adaptar la comunicación a las necesidades del paciente.

Por ello, es importante destacar la relación que hay entre comunicación y comportamientos/actitudes, es decir, cuidar las expresiones faciales y/o el lenguaje no verbal. Los investigadores ya han demostrado que es posible percibir las emociones de otra persona a través de expresiones faciales y cambios en la pigmentación de la piel, como por ejemplo el rubor y los gestos. Una expresión facial inapropiada puede afectar el comportamiento de muchas formas, ya que la comunicación no verbal es notable en las relaciones con los demás y en la forma en que se percibe a las personas. Por ende, esto puede tener un impacto significativo en el comportamiento y las interacciones sociales de las personas^(19)^.

Cuando se utiliza la escucha activa, la empatía y la comunicación asertiva, es pertinente hablar de receptividad. Los enfermeros emocionalmente inteligentes son mejores comunicadoras, tienen *más conductas de* cuidado^(20)^ y habilidades de adaptación para diferentes situaciones^(21)^.

Por consiguiente, la receptividad y la adaptabilidad están intrínsecamente ligadas a la CSE, ya que ambas son fundamentales para afrontar adecuadamente las interacciones sociales, los desafíos y los cambios en el entorno laboral y la vida personal. Por lo tanto, es necesario desarrollar estas competencias para tener interacciones sociales efectivas y bienestar emocional.

El entorno laboral en salud exige receptividad y adaptación a los nuevos retos y, todo el tiempo, los enfermeros toman decisiones asistenciales importantes. Por ende, se considera que hay receptividad cuando el profesional establece vínculos terapéuticos, promueve la confianza y la colaboración, y garantiza una atención de salud centrada en el paciente y la familia; la adaptabilidad se observa cuando el profesional tiene la capacidad de adaptarse a los cambios de forma equilibrada. Eso significa lidiar con la incertidumbre y la adversidad, lo que requiere control emocional^(22-24)^.

El comportamiento ético es esencial para la receptividad. La ética es una actitud fundamental que se vincula a la CSE de los enfermeros y profesionales de la salud de varias formas. Por lo tanto, además de las habilidades interpersonales, las CSE también abarcan cómo se relacionan los profesionales con los demás, cómo toman decisiones y demuestran respeto hacia los pacientes.

La postura ética en el trabajo del enfermero se conceptualiza como la capacidad de construir colectivamente procesos de trabajo, decidir éticamente, valorar la solidaridad, escuchar y compartir decisiones y gestionar conflictos, identificando los determinantes^(24)^. Por ende, los principios y valores éticos deben guiar las acciones y decisiones de los profesionales de enfermería, y garantizar la promoción, protección, recuperación y rehabilitación de la salud de las personas^(25)^.

El enfermero, al ser receptivo, también debe tener empatía. La empatía mejora la sensibilidad a las señales sociales que indican las necesidades de los demás, y esta competencia es particularmente importante en profesiones que brinda atención, como la enfermería^(26)^.

La adaptabilidad implica que el enfermero debe mostrar calma y paciencia cuando enfrenta diferentes problemas en su proceso de trabajo. Por ello, es importante señalar que, al actuar con calma, tolerancia, respeto hacia el equipo, tranquilidad, humildad y secretismo (confiabilidad), el profesional contribuye a la prestación de cuidados físicos, de apoyo emocional y a promover el bienestar general de los pacientes. Además, la empatía, el respeto, la calma y la paciencia se consideran componentes críticos para establecer una relación de confianza entre el equipo de enfermería, el paciente y el enfermero^(27-28)^.

Otra competencia incluida en la matriz fue el trabajo en equipo, que implica un enfoque en el que las personas colaboran y comparten responsabilidades, con el fin de lograr objetivos comunes. Los estudiosos también indican que, para que un grupo se convierta en un equipo, es necesario que haya algunos factores como compromiso, intercooperación y mayor conocimiento sobre el trabajo en equipo^(29-30)^.

Por ese motivo, el trabajo en equipo constituye una estrategia de integración de especialidades y múltiples profesiones, y es fundamental para el desarrollo de una asistencia y atención integral al paciente. Los atributos para este trabajo están vinculados a la comunicación interprofesional, al reconocimiento de otros profesionales y a los objetivos comunes del equipo en la interdependencia de acciones, la atención centrada en el paciente y la inteligencia emocional^(31)^.

Por lo tanto, para los enfermeros la inteligencia emocional es fundamental, dado que influye directamente en la calidad de la atención que les brindan a los pacientes, la capacidad de trabajar en equipo y el manejo de situaciones estresantes o desafiantes en el ambiente laboral. Articular la inteligencia emocional como CSE le permite al enfermero comprender profundamente las emociones propias y las ajenas, crear conexiones con los pacientes, comprender sus necesidades emocionales y brindarles apoyo profesional durante el proceso de tratamiento y recuperación^(32)^.

Siguiendo esta línea de pensamiento, otros investigadores mencionaron la relación que hay entre esta competencia y la conducta de profesionalismo, dado que forma parte de la promoción de la competencia emocional. Aquellos que poseen la capacidad de identificar emociones, utilizarlas en el proceso de pensamiento, comprenderlas, analizarlas y lidiar eficazmente con las suyas y con las de los demás son individuos emocionalmente inteligentes. La competencia emocional deriva del autoconocimiento y la autopercepción de la eficacia para controlar situaciones^(28)^.

Los comportamientos/actitudes vinculados a la inteligencia socioemocional se relacionan con poder distinguir entre lo profesional y lo personal, el autoconocimiento, el autocontrol y saber lidiar con la frustración y los conflictos. Se sabe que el trabajo del enfermero es complejo, involucra vínculos y proximidad con los pacientes, el equipo y las familias, por lo tanto, es fundamental que el profesional no permita que el trabajo interfiera en su vida personal. Además, es indispensable saber gestionar los conflictos de forma constructiva y empática.

Por ello, la gestión de los conflictos es otro factor relevante para el éxito de un equipo de salud, ya que puede afectar directamente la calidad de la atención que brinda. Los conflictos se consideran situaciones inherentes a la vida de los seres humanos, y pueden ser algo constructivo para el desarrollo personal. Por ese motivo, es el profesional quien debe gestionar los conflictos que se presentan o puedan ocurrir, para mantener motivado al equipo y que no afecten su productividad^(33-34)^.

Para el manejo asertivo de conflictos, el enfermero debe utilizar el liderazgo compartido, que también fue identificado como una competencia fundamental en la formación de los estudiantes. El liderazgo presupone saber delegar tareas y actividades. El enfermero en el proceso de trabajo gestiona el equipo, lo que puede sobrecargar de tareas a la persona que ocupa el rol de líder. Esa sobrecarga puede aliviarse si el gestor sabe delegarles actividades a los miembros de su equipo, y orientarlos para que resuelvan las diferencias de manera adecuada, sin infringir la cultura organizacional. El comportamiento de delegar tareas le permite al gestor tener mayor disponibilidad, analizar el potencial de los profesionales que están a su cargo y su compromiso y, además, construir una relación de confianza^(19)^.

La competencia del liderazgo compartido presupone comportamientos/actitudes relacionados con la postura, la gestión del tiempo, la educación continua y la distribución del personal. La postura física y la apariencia de los enfermeros se observa mucho y son necesarias para esta competencia. La forma en que los enfermeros se presentan físicamente puede afectar la forma en que la que los perciben los pacientes, los compañeros de trabajo y los equipos multidisciplinarios y, a su vez, puede influir en las interacciones sociales y emocionales^(28)^. Por ello, es importante concienciar a los profesionales para que adopten una postura y apariencia agradable, un comportamiento servicial y usen ropa limpia y adecuada al entorno laboral.

Otro comportamiento asociado al liderazgo compartido es la gestión del tiempo. Tener la capacidad de gestionar el tiempo de manera efectiva mejora la productividad y contribuye a establecer relaciones saludables y a tener equilibrio emocional^(35)^.

Los estudiosos encontraron pocas estrategias para que los estudiantes de enfermería desarrollen las CSE. En los hallazgos de este estudio, se observó que había poca o casi ninguna orientación por parte de profesores o profesionales especialistas en el área con respecto al control de las emociones durante el cursado de la carrera de enfermería. El plan de estudios de la carrera no presenta contenidos, asignaturas o actividades que apunten a este tema.

En el desarrollo de la investigación, la realización del GF en sí fue una estrategia para valorar el abordaje socioemocional con los estudiantes y provoca la movilización de las CSE en los participantes. El GF es un método de investigación colaborativa que involucra a los participantes en la construcción activa del conocimiento, y su enfoque explora la interacción entre el lenguaje, la subjetividad y la construcción social de la realidad. Esto demuestra que se pueden adoptar recursos simples y breves en la carrera de grado, para crear procesos de desarrollo de las CSE^(36)^.

Por lo tanto, se observa que aumentar la inteligencia emocional de los profesionales de la salud debería ser una preocupación exponencial. Centrarse únicamente en las habilidades técnicas no garantiza el éxito profesional de los enfermeros, y con el aumento de la tecnología, las habilidades no técnicas serán el factor diferenciador entre los profesionales de la salud.

Crear un ambiente de aula equilibrado, armonioso y propicio para el aprendizaje de valores y contenidos disciplinarios se ha convertido en un gran desafío constante y requiere que el docente cuente con diferentes conocimientos y competencias, como es el caso de las CSE^(37)^.

Se pueden enumerar algunas estrategias específicas, como: grupos de apoyo para problemas emocionales; Programas de Educación Tutorial; estímulo a través de la investigación; terapia; simulaciones de carácter emocional; ligas académicas; tutorías sobre planificación de la carrera, entre otros. Estos datos coinciden con los de otros investigadores que han identificado varias estrategias que promueven el desarrollo de competencias personales y sociales, tales como: aprendizaje reflexivo grupal; actividades experienciales; profundizar las competencias de inteligencia emocional; simulaciones; investigación; y otros. Si bien el uso de las estrategias identificadas promueve la inteligencia emocional, este proceso no es estanco y requiere capacitación y análisis constante de la práctica del enfermero, con el fin de mejorar su comportamiento a través de la mejora de las competencias personales y sociales^(38-39)^.

Este trabajo define lo importante que es elaborar una matriz de CSE con sus factores asociados, comportamientos/actitudes, al vislumbrar que es necesario incluir ese tema en los proyectos académicos de las instituciones de formación en enfermería, ya sea en las carreras de grado o de posgrado *lato* y *stricto sensu*. Elaborar una matriz de CSE específica para los enfermeros es una iniciativa muy importante, dado que se sabe lo relevantes que son las habilidades emocionales y sociales en la práctica de enfermería^(40)^.

Esta investigación tiene una limitación y es que se realizó solamente en una IES pública, y no incluyó el análisis de otras instituciones de formación. Además, esta investigación se realizó exclusivamente con la participación de estudiantes del último año de enfermería. Es importante que se realicen otros estudios, con el fin de abarcar otras instituciones educativas, incluso las privadas, además de profesionales graduados, docentes u otras categorías profesionales, con el objetivo de verificar si existen divergencias y/o determinar hasta qué punto se pueden generalizar los hallazgos.

Este trabajo revela una matriz de CSE que puede realizar varias contribuciones significativas al avance del conocimiento científico en el campo de la educación y formación de enfermería, así como también para incorporar las CSE en el currículo académico de las universidades.

## Conclusión

Al cumplir con el objetivo de este estudio, se pudo crear una matriz de CSE para estudiantes de grado en enfermería. Para cada competencia identificada, el estudiante debe tener actitudes/comportamientos específicos. Proporcionar una matriz con esta información sobre una categoría profesional puede contribuir positivamente a la formación de los estudiantes y beneficiar a los trabajadores.

La reflexión sobre cómo es la formación y preparación de los futuros enfermeros con respecto a las habilidades sociales y emocionales hará la diferencia en el desarrollo de la inteligencia emocional de esas personas. Pensar en las emociones mejora la capacidad de autoconocimiento. Además, al caracterizar las CSE a través de la percepción de los futuros enfermeros, les permite formarse en la gestión del clima laboral y promover su propio bienestar y el de su equipo. Invertir en el desarrollo socioemocional de los estudiantes de enfermería tiene el potencial de elevar el nivel de atención que se brinda y preparar profesionales resilientes capaces de enfrentar los desafíos de la práctica clínica con compasión y competencia. Por lo tanto, la creación de esta matriz sobre CSE para estudiantes de enfermería debe ayudar a las instituciones de formación a elaborar proyectos pedagógicos orientados a la formación basada en competencias. Además, tiene el potencial de cubrir posibles vacíos en el desarrollo profesional de los futuros enfermeros y prepararlos con un perfil de CSE que también esperan las organizaciones de salud y los pacientes.
